# Types of Relational Aggression in Girls Are Differentiated by Callous-Unemotional Traits, Peers and Parental Overcontrol

**DOI:** 10.3390/bs5040518

**Published:** 2015-11-13

**Authors:** Luna C. M. Centifanti, Kostas A. Fanti, Nicholas D. Thomson, Vasiliki Demetriou, Xenia Anastassiou-Hadjicharalambous

**Affiliations:** 1 Department of Psychology and Wolfson Research Institute, Durham University, Durham DH1 3LE, UK; E-Mail: n.d.thomson@durham.ac.uk; 2 Department of Psychology, University of Cyprus, Nicosia 1678, Cyprus; E-Mail: kfanti@ucy.ac.cy; 3 Department of Psychology, University of Nicosia, Nicosia 1700, Cyprus; E-Mails: demetriou.v@student.unic.ac.cy (V.D.); hadjicharalambous.x@unic.ac.cy (X.A.H.)

**Keywords:** callous-unemotional traits, aggression subtypes, parent-child relationship, peers, females

## Abstract

Adolescent girls often perpetrate aggression by gossiping and spreading rumours about others, by attempting to ruin relationships and by manipulating and excluding others. Further, males and females engage in reactive and proactive relational aggression differently. In this study, we examined the individual, peer and parental contextual factors that best explained the use of reactive and proactive relational aggression in girls. Female participants (*n* = 614; ages 11–18 years) completed questionnaires on aggression, callous-unemotional (CU) traits, delinquency, peer delinquency, gender composition of their peer group, resistance to peer influence and perceived parental overcontrol. Multinomial logistic regression was used to examine the effects of individual, peer- and parent-related variables on the likelihood of being classified as a low aggressor, reactive aggressor or proactive/reactive aggressor. Girls in the combined reactive/proactive aggression group were younger, had greater CU traits, a lower proportion of male peers and greater perception of parental control than both the reactive and low aggressive groups. Both highly aggressive groups were more delinquent and had greater peer delinquency than the low aggressive group. This study suggests those girls who show relational aggression for the purpose of gaining status and revenge feel restrained by their parents and may gravitate toward relationships that support their behaviour.

## 1. Introduction

Adolescent girls often perpetrate aggression by gossiping and spreading rumours about others, attempting to ruin relationships and manipulating and excluding others. For the past few decades, research has also looked at the differences between people who use physical forms of aggression and those who use more indirect or relational forms of aggression [1,2]. Although girls and boys may not differ in the overall use of relational aggression [3], they may differ in how damaging this use of aggression is to their peer relationships [4]. Girls who used high levels of relational forms of aggression showed the worst adjustment problems; this was notwithstanding the level of physical aggression they displayed [2]. For girls, then, a high use of relational aggression for multiple purposes (retaliatory and for personal gain) may demarcate girls who have problems maintaining satisfying and prosocial relationships with others. For example, girls who used high levels of relational aggression showed low levels of caring and empathy toward others, characteristics associated with a callous-unemotional (CU; *i.e.*, lack of remorse or empathy, callous use of others, shallow or deficient emotions) interpersonal style [2,5]. Adolescent girls who gossip and work to ruin relationships by freezing people out may show adjustment problems, but also, this may depend on their reasons for using relational aggression. Relational aggression, on its own, may be particularly important to look at in girls, because these aggressive tactics appear to negatively affect girls more than they affect boys.

The “why” of aggressive behaviour has been important in research on gender differences [2,4,6]. That is, girls may engage in relational aggression for different reasons, ranging from reactive aggression that is in response to a real or perceived slight to proactive aggression that is done for personal gain or to obtain some desired outcome, such as status (e.g., popularity) or a desired object [7,8]. Those who show high levels of reactive aggression, but not proactive aggression, are emotionally reactive and show high levels of social-cognitive biases, such as interpreting unclear, but negative behaviours enacted by others, as being malicious [8,9,10]. People with high levels of reactive aggression also tend to be impulsive and have problems implementing adaptive emotional and behavioural regulation strategies [5,9,11]. In contrast, those who use proactive aggression tend to engage in planned and controlled aggressive behaviour and show blunted emotion; or they may show emotion that is inconsistent with their behavioural displays [12,13,14].

With regard to relational aggression, females who used high levels of reactive aggression had a strong tendency to perceive others actions as hostile and malicious, yet this was not shown for males [15]. Thus, examining reactive and proactive aggression within relational aggression and the association with adaptive functioning in girls may shed light onto the important processes involved in girls’ aggression. By looking at factors known to relate to aggression for adolescent girls, such as individual characteristics, as well as environmental factors, we aim to examine the factors most associated with relational aggression for girls.

Adolescents who tend to show high levels of proactive aggression also tend to show high levels of reactive aggression. In fact, the two subtypes show high correlations. Recent research then uses clustering techniques based on covariations between reactive and proactive aggression [2,5,14]. Prior research has identified a group of adolescents that displays high levels of both proactive and reactive aggression and a group that displays a high level of reactive aggression, but relatively average levels of proactive aggression. Typically, a low aggressive group, relative to the other groups, is identified. Cluster analyses have revealed similar groupings for relational aggression, specifically [2]. As would be predicted, youths who use aggression to retaliate for some perceived provocation, as well as to gain favours over others display cold, uncaring and callous behaviours [2,5,16]. Thus, a small, but significant sample of youths may be identified who show a combined form of aggression and can be differentiated by levels of CU traits.

## 2. Individual-Level Factors

People with CU traits have been found to show a combined form of aggression that includes proactive and reactive aggression [2,14,17]. Marsee *et al.* [2] examined the role of CU traits in relational aggression. They examined the forms and functions of aggression in their study of community, detained and residential boys and girls (ages 11–20 years). Findings revealed that relationally-aggressive girls showed high rates of delinquency and high levels of CU traits. Indeed, the highest levels of CU traits were shown for the group of girls with a combination of high reactive and proactive relational aggression [2]. This has also been shown in a detained sample of girls, where the combined reactive/proactive relational group of girls was highest on CU traits [5]. Thus, there may be particular factors associated with engaging in relational aggression for girls that sets them on the road to further maladaptive functioning. Relational aggression may, therefore, be a marker for girls’ problematic behaviour.

## 3. Peer-Level Factors

Social contextual factors have begun to be examined in relation to reactive and proactive aggression, and these may be particularly important for distinguishing reactive and proactive aggression subtypes [18,19]. Further, we may be able to identify factors that, from prior research, are predictors of aggression in adolescent girls; these factors may explain the combination of reactive and proactive relational aggression. In both males and females, having important peers that are predominantly male is related to greater engagement in antisocial behaviour [20]. For example, they report doing more delinquent acts, such as getting drunk, shoplifting and vandalizing property, with boys than with girls [21]. Of importance, adolescent females who report having more other-sex friends perpetrated more severe violence, and the level of violence increased proportionally with the number of other-sex peers [22]. Girls’ closer friendships may allow for proactive relational aggression to be used to exploit their relationships for personal gain [23].Thus, having a greater proportion of males in one’s peer group may relate to the use of high levels of aggression and possibly a combined type of aggression. However, it is possible that all-female peer groups facilitate relational aggression strategies that are done for instrumental reasons (to pursue a goal).

Youths who engage in antisocial behaviour tend to have similarly antisocial peers. Indeed, hanging out with antisocial peers designated a group of females who engaged in antisocial behaviour throughout childhood and adolescence [24]. In addition to peer socialization processes explaining youths’ association with antisocial peers, youths may select peers who mirror their own normative beliefs about aggression and risky behaviour [25]. Some of the influence exerted by peers may be explicit: friends may directly embolden youth and reinforce their antisocial behaviours [26,27]. However, peers also may implicitly exert influence *vis*-*à*-*vis* contagion through competition, status enhancing norms and other social-cognitive mechanisms [28,29,30]. Adolescents’ perceptions of their peers’ delinquency may, therefore, relate to their own aggressive behaviour, since perpetration of relational aggression is facilitated by friends that are complicit in freezing others out. Only one study to our knowledge has examined peer delinquency and subtypes of aggression. This study found bidirectional effects only between reactive aggression and peer delinquency [31]. However, Fite and Colder [31] did not use person-centred analyses, such as clustering, which could explain a lack of significant association with proactive aggression in their study. That is, they examined relations with proactive aggression while controlling for reactive aggression, yet some have questioned this statistical technique as partialling out true variance and leaving mainly error variance. Fite and Colder also did not measure relational aggression. Since personality characteristics that have been found to relate to a combined proactive and reactive relational aggression are also related to hanging out with antisocial peers [32], it may be that peer delinquency will be higher for the combined reactive and proactive relational aggressive group of girls than for the other relationally-aggressive groups of girls.

As adolescents mature, they are better able to resist the influence of peers; this seems to relate to increases in cognitive maturity [33]. Further, Steinberg and Monahan [33] argue that the change in resistance to peer influence observed from the ages of 14–18 years likely reflects individuation from parents at the same time as seeking greater involvement with peers and managing these peer relationships. As a result of this literature, and given that adolescent risk-taking almost always occurs in groups, some scholars speculate that the presence of peers stimulates adolescent antisocial behaviour by increasing the saliency of potential short-term rewards [34]. For example, Steinberg’s biobehavioural model suggests that having peers in the vicinity automatically triggers the activation of reward processing centres of the brain and incites adolescents toward greater risky decision making [34,35]. As youths age and enter adulthood, the rewarding aspects of peer influence become dampened by inhibitory and executive processes in the brain, which manage and modulate emotions. Thus, examining resistance to peer influence as another social contextual factor related to aggressive behaviour is needed, since proactive aggression has been particularly associated with increased sensitivity to rewards.

## 4. Parent-Level Factors

Some adolescent girls may seek to individuate themselves from their parents due to the desire to be independent and, thus, seek to be free of their parents’ control [36]. According to self-determination theory [37], for example, basic needs include psychological autonomy and relatedness. Adolescents may perceive parents’ actions as hindering the fulfilment of autonomy, which they see as affecting the development of close relationships with their peers. If adolescent girls perceive their parents as exerting excessive control, they may feel that their autonomy-seeking is being blocked [36]. In response, they might choose to engage in activities where adults are absent and where their activities with peers go unmonitored. If girls pursue unstructured activities with peers where adult supervision is lacking, they may encounter more opportunities to engage in antisocial behaviour and aggression [38]. However, in at least one study, parental control was not as predictive of adolescent behaviour as was peer delinquency [39,40]. In another study, girls who had an early start to their antisocial behaviour more often reported having problems with their parents [41]. Thus, it may be that the perception of the parent-child relationship [42] is important in distinguishing the “why” of relational aggression, particularly for girls.

## 5. Present Study

The primary aim of the present study was to differentiate subtypes of aggression based on factors related to adolescent girls’ maladaptive behaviour, including social contextual factors that have not typically been included in research on reactive and proactive relational aggression. Multiple contextual factors (*i.e.*, parents and peers) were included to elucidate the potential processes involved in reactive and proactive relational aggression. Thus, we argue that by including measures beyond individual-level factors, we may be able to show support for the assessment of subtypes of aggression.

In a large sample of high school girls from Cypriot schools, we examined individual-level characteristics consisting of delinquency, substance use and CU traits predicting relational aggression, while classifying girls by their use of reactive and proactive relational aggression. This is shown in Table 1. Lansford and colleagues [4] found that relational aggression showed similarities in gender associations across cultures. In this study, we examined social contextual effects of peers and perception of parental overcontrol as additional factors over and above the effects of the individual, which is consistent with prior research examining contextual factors over individual-level factors [40]. Further, we tested variations between groups on individual-, peer- and parent-level variables to see whether adolescent girls with distinct profiles of aggressive behaviour differ. To examine reactive and proactive aggression, we used cluster analysis to identify the hypothesized combined reactive/proactive group, a primarily reactive group and a low aggressive group. After including the individual-level predictors, we hypothesized that peer and parent factors would differentiate the two aggressive groups, such that those in the combined group were predicted to be less resistant to peer influence, have a greater proportion of male peers, perceive parents as being more controlling, in addition to reporting the highest levels of CU traits, as compared to the reactive-only and low aggressive groups. We explored whether both aggressive groups were higher on peer delinquency than the low aggressive group.

**Table 1:** Data analysis plan in predicting reactive and proactive relational aggression clusters. CU, callous-unemotional.

Predictors	Dependent Variable: Clusters
Individual Factors	
	Use of drugs
	Delinquency
	CU traits
Peer Factors	
	Peer delinquency
	Male peers
	Resistance to peer influence
	Romantic partner
Parenting Factor	
	Freedom from parental overcontrol

## 6. Method

### 6.1. Participants and Procedure

The sample consisted of a community sample of 614 girls (aged 11–18). The schools were chosen to approximate the national demographics in Cyprus. Almost all of the girls were Greek Cypriot (92%). About half of the parents reported having a high school (55% for fathers, 48% for mothers) or a college/university education (25% for fathers, 32% for mothers).

Ethical approval, school approval and parental written informed consent were obtained before participation in the study; children’s participation was voluntary. Parents were contacted through the schools by sending a briefing sheet with a consent form for parents to tick “yes” or “no” to participation. Only if parent(s) agreed, children were approached for their assent to engage in the research. The rate of response was 60%, which is considered satisfactory for this kind of study given that no incentive was offered for participation. Importantly, less than 1% of the approached parents actively dissented. The remaining failed to return a consent form, and therefore, it remains unknown whether the parents of these children did not want their child to participate or whether they had not read the consent form.

### 6.2. Measures

Peer Conflict Scale: The Peer Conflict Scale (PCS) [43] is a Likert-type questionnaire to be completed by the participant. The questionnaire includes 40 items, with each item scored from 0 = not at all true to 3 = definitely true. The PCS was developed to overcome the limitations of previous measures of reactive and proactive aggression. Specifically, the proactive subscale was broadened to include not only aggression for gain, but also aggression for dominance (e.g., “I gossip about others to become popular”), aggression for sadistic reasons (e.g., “I enjoy making fun of others”) and unprovoked and premeditated aggression (e.g., “I spread rumours and lies about others to get what I want”). The reactive subscale was also expanded to include not only emotionally-provoked, angry aggression, but also impulsive, thoughtless aggression (e.g., “When I have started rumours about someone, it is usually because I acted without thinking”). The PCS is considered a reliable measure for instrumental aggression and aggression types with good internal consistency [43]. For the present study, we used the 20 relational aggression items, of which 10 measured reactive relational aggression and 10 measured proactive relational aggression. Internal consistency for the present study was good and is listed in Table 2.

Delinquency/drug use: To gather data about the female participants’ delinquency, the Self-Report of Delinquency (SRD) was used. The SRD consisted of 29 items from the Elliott and Ageton [44] self-report inventory, including asking for the age at which they first engaged in the act. The items used were property offences, drug offences, status offences and items regarding violent offences. A study reviewed the literature comparing self-reported delinquency with official records of delinquency and concluded that both ways of measuring delinquency “provide valid indicators of the demographic characteristics of offenders” ([45], p. 995); this research gives support to the reliability and validity of self-report measures. The severity of the delinquency was measured by the mean number of items that were endorsed. Therefore, we chose to use a variety measure of delinquency rather than a frequency measure. Variety scores are typically used to assess criminal activity [46,47] and are strongly related to frequency scores [25]. However, variety scores have an obvious benefit in that it is much easier to remember if one has engaged in an illegal activity than it is to remember the frequency. This is especially the case with activities that tend to occur with greater frequency, such as drug offenses [25].

**Table 2:** Correlations among the main study outcomes.

	1	2	3	4	5	6	7	8	9	10	11
1. Reactive aggression											
2. Proactive aggression	0.66 **										
3. Age	−0.06	−0.13 **									
4. Use of drugs	0.14 **	0.09 *	0.21 **								
5. Delinquency	0.31 **	0.23 **	0.23 **	0.56 **							
6. CU traits	0.21 **	0.31 **	0.08	0.27 **	0.34 **						
7. Peer delinquency	0.29 **	0.22 **	0.12 **	0.58 **	0.66 **	0.24 **					
8. Male peers	0.11 *	0.04	0.17 **	0.25 **	0.25 **	0.06	0.33 **				
9. Resistance to peer influence	−0.10 *	−0.13 **	0.11 **	0.04	−0.01	−0.20 **	−0.03	0.09*			
10. Romantic partner	0.04	0.06	0.24 **	0.23 **	0.29 **	0.06	0.26 **	0.50 **	0.06		
11. Free from parental overcontrol	−0.20 **	−0.20 **	0.15 **	−0.01	−0.04	−0.10 **	−0.07	−0.03	0.05	0.01	
*Descriptive statistics*											
Mean (SD)	4.50 (3.73)	1.84 (2.96)	15.85 (1.65)	0.64 (1.29)	4.28 (4.13)	17.54 (7.50)	0.44 (0.49)	0.70 (0.22)	26.66 (7.82)	0.31 (0.46)	9.85 (2.44)
Cronbach’s alpha	0.71	0.79	N/A	0.48	0.85	0.74	0.88	N/A	0.68	N/A	0.80

Notes: * *p* < 0.05; ** *p* < 0.01.

Drug use was assessed using three items. These items were chosen because they assess the frequency of use, rather than general use or trafficking, which is measured using the Self-Report of Delinquency. These items have been included in studies of norm-breaking [48], where they have been shown to be associated with keeping secrets from parents and feeling overcontrolled by parents. In another study, norm-breaking, including the two drug items, was related to youth involvement with boys in unstructured settings, such as in youth community centres [38]. Therefore, the items were of interest to the present study, in addition to the Self-Report of Delinquency. The items asked “Have you drunk so much alcohol (beer, liquor, wine) that you got drunk”, “Have you smoked marijuana-hashish (pot, grass, cannabis, weed)?” and “Have you used any drugs other than marijuana-hashish (pot, grass, cannabis, weed), other than prescribed for you?” The five response options ranged from “No” (0) to “More than 10 times” (4).

Peer delinquency: Peer delinquency was derived from the Peer Delinquent Behavior scale [49], which assesses peer delinquent behaviour by asking generally about youths’ friendships rather than particular friends. Participants responded to 12 items, which were pertinent to their peers’ antisocial behaviour. Items included “How many of your friends have sold drugs?” Participants responded on a 5-point Likert scale ranging from “None of them” to “All of them.” Research has found composites including this measure were related to the target’s own delinquency and for the association between own and peer delinquency to change across developmental periods indicating peer selection and influence effects across adolescence [25].

Inventory of callous-unemotional traits: Participants completed the 24-item Inventory of Callous-Unemotional traits (ICU) [50]. This rating scale is rated on a four-point Likert scale indicating 0 “not at all true” to 3 “very true”. The ICU has been validated in a community sample (*n* = 1443) of German adolescents ages 12–18 [51], a school-based sample (*n* = 347) of Greek Cypriot adolescents ages 12–18 [52], a school-based sample (*n* = 455) of adolescents ages 14–20 in Flanders, Belgium [53], and a moderate-sized (*n* = 248) sample of juvenile offenders ages 12–20 in the United States [54]. A similar factor structure has emerged across studies with three factors (e.g., uncaring, callousness, unemotional) loading on a higher-order CU dimension. Of importance, the total scores proved to be internally consistent in these samples (Cronbach’s alpha ranging from 0.77–0.89), and they were related to antisocial behaviour, aggression, delinquency, various personality dimensions and psychophysiological measures of emotional reactivity in ways consistent with past research on CU traits. Internal consistency was good and is listed in Table 2.

Peer nomination: The participants were asked to list the age, gender and relationship of 10 important peers using the “very important persons” measure used before [55]. This peer nomination measure has been used in prior studies [56], and prior research shows nominations have been found to be reciprocated even for antisocial youths [56]. The relationship choices included “friend”, “sibling”, “cousin”, “romantic partner”, “online” or “other”. The proportion of males in the friend, romantic partner and online relationship categories was computed for each participant.

Resistance to peer influence: The Resistance to Peer Influence measure (RPI) [33] asked participants to respond to a series of paired statements designed to measure resistance to peer influence in general, not only to antisocial influence from peers. The RPI consists of 10 paired statements, including “Some people go along with their friends just to keep their friends happy”, but “Other people refuse to go along with what their friends want to do, even though they know it will make their friends unhappy”. Participants were then given the option to select the statement that was the best descriptor for them. After they chose the best descriptor, they further indicated how much the descriptor fit them by choosing one of “really true” or “sort of true.” The responses were coded on a 4-point scale, with the scale ranging from “really true” for one statement/descriptor to “really true” for the other statement. The scores were then summed, and higher scores indicated greater resistance to peer influence. Steinberg and Monahan showed the validity of this measure with regard to peer delinquency [25], antisocial behaviour, age-related changes through childhood and adolescence (possibly related to seeking independence from parents and relying more on peers) and patterns of neural connectivity [33]. Internal consistency was acceptable in the present study (see Table 2).

Freedom from parental overcontrol: The free from parental overcontrol measure consisted of five items that examined youths’ feelings of being free from parental overcontrol, with options ranging from “no, never” (5) to “yes, always” (1). Items included “Do you think that your parents give you enough freedom to do what you want during your free time?” and “Do you think that your parents control everything in your life?” (reversed). Research has found this measure to be related to feeling connected with parents and to children’s willingness to self-disclose [36] and has shown good test-retest reliability over a two-month period [48]. See Table 2 for descriptions.

Translation of instruments: The English version of the measures were adapted and translated according to guidelines that are widely accepted for the successful translation of instruments in cross-cultural research [57]. One bilingual translator who was also a native speaker or culturally-informed individual blindly translated the questionnaires from the original language (English) to the second language (Greek), and another bilingual person translated it back to the original language. Differences in the original and the back-translated versions were discussed and resolved by joint agreement of both translators.

## 7. Results

### 7.1. Creating Aggressive Subtypes

A two-step cluster analysis was performed using SPSS 20 to create groups of low aggressive, reactive aggressive and combined aggressive girls; this is similar to prior research on aggression subtypes [2]. The reactive and proactive aggression subscales from the PCS were standardized prior to analyses. The two-step method is an auto-cluster procedure, which combines information from both Bayesian information criteria (BIC) and the ratio of the distance between clusters to determine the optimal number of clusters to retain. Clustering, using the two-step procedure, is based on a probabilistic model where the distance between clusters is parallel to the decrease in the log-likelihood function, which is a result of merging nearest neighbours [58]. First, pre-clusters are formed based on a sequential approach where pre-clusters are formed when the log-likelihood is maximized. A likelihood distance measure is used to determine each case’s similarity to an existing pre-cluster. Similar to agglomerative hierarchical procedures, the second step uses a model-based hierarchical clustering. The statistical program determines the number of clusters by weighing both the ratio of the distance between clusters and the change in BIC, such that a decrease in BIC from a previous model suggests a better fit. The silhouette coefficient of cluster separation (distance of cases from the next closest cluster) and cohesion (distance of a case from the centre of its own cluster) was examined as a fit indicator for the resulting clusters. This coefficient ranges from −1 (poor fit) to one (excellent fit) [59].

**Figure 1:**
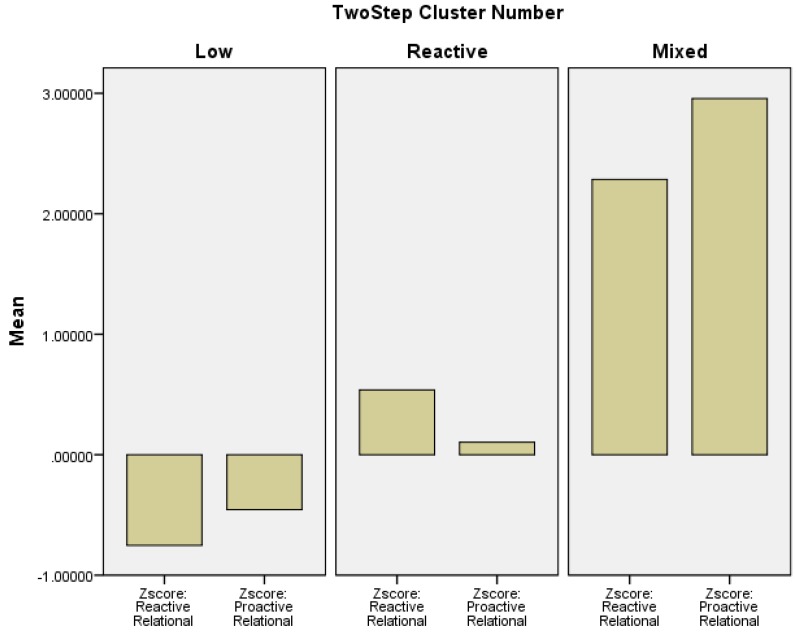
Profiles of reactive and proactive relational aggression resulting from two-step cluster analysis.

A three-cluster model was selected as fitting the data best; this was a good fitting model according to the silhouette coefficient (0.6). The ratio of the distance between clusters was 2.34 as compared to 1.64 and 1.83 for the two-cluster and four-cluster solutions tested, respectively. Furthermore, the ratio of BIC changes showed a bigger change (0.58) from two (BIC = 577.17) to three (BIC = 406.04) clusters than the change (0.20) from two to four (BIC = 347.82) clusters. The profile of the three clusters is provided in Figure 1. Consistent with predictions, there was a low aggression cluster (*n* = 307, 50%), a cluster relatively high on reactive aggression (*n* = 268, 44%) and group high on both reactive and proactive aggression (combined cluster; *n* = 38, 6%). As noted in Figure 1, the combined cluster showed the highest levels of both reactive and proactive aggression.

### 7.2. Effects of Individual and Environmental Factors on the Likelihood of Being Classified as a Low, Reactive or Proactive/Reactive Combined Aggressor

Table 2 lists the zero-order correlations. Reactive and proactive aggression were significantly and positively correlated with delinquency, use of drugs, CU traits and peer delinquency. Both were negatively correlated with resistance to peer influence and feeling free from parental overcontrol. Further, proactive aggression was inversely related to age, and reactive aggression was positively related to the proportion of male peers. As expected, CU traits were related to the use of drugs, delinquency and peer delinquency, but were negatively related to resistance to peer influence. Further, CU traits were negatively related to feeling free from parental overcontrol. Delinquency and peer delinquency were positively and highly correlated with each other (*r* = 0.66), suggesting overlap in self-reporting and perception of peer delinquency. Both delinquency and peer delinquency were related to use of drugs, having a greater proportion of male peers and having a romantic partner in the important peer group. Having a greater proportion of male peers and having a romantic partner were associated with greater use of drugs and greater resistance to peer influence, which was unexpected.

Hierarchical multinomial logistic regression analysis was used to investigate the unique effects of individual, peer- and parent-related variables on the likelihood of being classified as a low aggressor, reactive aggressor or proactive/reactive aggressor. The first step included age, use of drugs, delinquency and CU traits. The second step of the logistic regression included the main effects of peer-related variables: peer delinquency, proportion of male peers, resistance to peer influence and having a romantic partner. The third step of the logistic regression included the parental overcontrol variable. Results are shown in Table 3. Odds ratios are incorporated to compare the different groups. In general, odds ratios reflect the odds likelihood of being in one group over the other, on the basis of the level of the independent variable.

**Table 3:** Multinomial logistic regression analysis.

	Group Comparisons Based on Odds Ratios (95% CI)		
	Proactive/Reactive *vs.* Low	Reactive *vs.* Low	Proactive/reactive *vs.* Reactive
Step 1			
Age	0.61 ** (0.49–0.77)	0.93 (0.83–1.03)	0.66 ** (0.53–0.83)
Use of drugs	0.85 (0.63–1.15)	0.92 (0.77–1.09)	0.93 (0.71–1.23)
Delinquency	1.25 ** (1.14–1.38)	1.18 ** (1.11–1.26)	1.06 (0.98–1.16)
CU traits	1.12 ** (1.07–1.18)	1.01 (0.98–1.04)	1.11 ** (1.06–1.17)
Step 2			
Peer delinquency	3.28 * (1.14–9.45)	2.08 * (1.13–3.85)	1.58 (0.60–4.15)
Male peers	0.11 ** (0.02–0.64)	0.66 (0.26–1.68)	0.17 * (0.03–0.92)
Resistance to peer influence	0.98 (0.93–1.03)	1.01 (0.98–1.03)	0.97 (0.93–1.02)
Romantic partner	0.70 (0.27–1.78)	0.93 (0.60–1.44)	0.75 (0.30–1.85)
Step 3			
Freedom from parental overcontrol	0.86 ** (0.79–0.94)	0.99 (0.95–1.03)	0.87 ** (0.80–0.95)

* *p* ≤ 0.05; ** *p* ≤ 0.01.

The first step of variables had a significant impact on the model fit, *x*^2^(8, *n* = 601) = 91.75, *p* < 0.001. Girls in the combined proactive/reactive group had a greater likelihood of being younger in age in comparison with the reactive and low aggression groups. Girls who were classified as reactive or combined aggressors were more likely to engage in delinquency compared to the low aggression group. Girls classified as combined aggressors scored higher on CU traits compared to both the reactive and low aggression groups.

The inclusion of the main effects of peer variables in the second step of the multinomial logistic regression improved the model fit, *x*^2^(8, *n* = 601) = 20.07, *p* = 0.01. Girls who reported higher peer delinquency were more likely to be classified in the reactive and combined groups as compared to the low aggression group, and girls with lower proportions of male peers were more likely to be classified in the combined group compared to the reactive and low aggression groups. Levels of resistance to peer influence and romantic partners did not differentiate the odds of being a reactive aggressor *versus* a combined proactive/reactive aggressor.

The addition of the perceived freedom from parental overcontrol measure in Step 3 also had an impact on the model fit, *x*^2^(2, *n* = 601) = 10.61, *p* < 0.01. The findings suggested that girls in the combined group were less likely to perceive greater freedom from parental overcontrol than girls in the reactive and low aggression groups.

## 8. Discussion

Consistent with prior research, our findings suggest that the individual and peer delinquency levels did not significantly differentiate the two aggressive groups, indicating that they are not distinct on behavioural factors. Therefore, although the combined aggressive group was higher on aggressiveness, it was not differentiated from the reactive aggressive group based on delinquency. However, our findings suggest that individuals with both reactive and proactive aggression show different peer-level and parent-level correlates compared to those in the predominantly reactive aggressive group [1,13,60]. Those in the combined aggressive group were differentiated from the reactive group by being younger, having higher CU traits, having a lower proportion of male peers (in relation to females) and perceiving parents as being more controlling. Thus, we were able to show differentiation across aggressive subtypes when including factors beyond the individual’s behaviour.

Research has not previously examined the gender composition of peer groups in relation to subtypes of aggressive behaviour, as far as we know. In prior research, physically-aggressive girls have been shown to be rejected by their female peers due to their deviation from the gender norm, and they gravitate more towards male-dominated peer groups [61]. However, higher peer status and popularity have been shown to be related to girls’ use of relational aggression [62], indicating a possible divergence from prior research based on the form of aggression. Thus, it may be that female peers are accepting and possibly attracted toward associating with highly relationally-aggressive and more proactive aggressive girls. Although these girls may not be liked, they may still attract high peer status [62]. Alternatively, it may be that this combined group was lower in reporting many more male peers because this group was younger. In the zero-order correlations, older girls associated with a greater proportion of male peers. Moreover, in our models, having a romantic partner identified in the important peer network did not differentiate the groups. Yet, in the zero-order correlations, reactive aggression, delinquency, peer delinquency and drug use were significantly related to reporting a greater proportion of male peers in the peer group, as well as having a romantic partner within the important peer network (except for aggression). Thus, this finding requires further research to determine whether the gender composition of peer groups yields useful effects beyond other measures of peer behaviour.

A further differentiation for the combined reactive/proactive aggressive group was revealed when including perceived parental overcontrol. In particular, perceiving parents as exerting too much control differentiated the combined aggressive group from the reactive and low aggressive groups. Although this group was younger, they were eager to “…(knife)-off childhood apron strings…” ([63], p. 688) by possibly seeking to be less controlled by their parents. That is, they were less likely to report feeling their parents were giving them their freedom when compared to the other two groups. Thus, beyond the individual- and peer-level measures, the proximal environment of the parent-child relationship (as perceived by girls) specifically differentiated the types of aggressive behaviour and was not simply related to severity of aggression. Of note, in the zero-order correlations, CU traits were related to perceiving parents as not providing enough freedom from overcontrol. Consistent with research on youths with psychopathic traits (including CU traits), youths high on CU traits may perceive their proximal social network as failing to be supportive of their behaviour [56].

Of importance, we found that CU traits significantly differentiated the combined aggressive group from the reactive aggressive and low aggressive groups. These findings with CU traits are consistent with prior research [2,5,52] that have examined the role of CU traits in aggression. The present study extends prior research by replicating these findings in a Cypriot sample of community girls. This difference across reactive and combined types suggests that CU traits designate differences across aggressive types and does not simply vary with severity of aggression, which could have been argued given that our two highly-aggressive groups may be perceived to differ on both reactive and proactive aggression in an incremental way. However, when looking at other individual-level measures, such as emotional and socio-cognitive factors, prior research found support for a severity effect rather than a difference across subtypes of aggression [2,5]. Yet, like these other studies, the present study found that CU traits were the only individual-level measure that differentiated the two highly-aggressive groups, suggesting that the relatively predominant reactive aggressive profile differs from the combined reactive/proactive profile of aggression.

Both highly-aggressive groups were differentiated from the low aggressive group by their higher levels of delinquency and perceived peer delinquency. The link between reactive and proactive aggression and delinquency has a robust history in the research literature [64,65,66]. Fite and colleagues [64] find prospective associations over time, such that proactive aggression predicts delinquency one year later. Our findings suggest that delinquency is related to both types of relational aggression, possibly demarcating mixed relational aggression in girls as a marker for “...extreme overall level of disturbance...” ([5], p. 523). Further, it is unsurprising that those in the combined aggressive group were higher on peer delinquency, given that they were higher on delinquency and were differentiated by higher levels of CU traits. Prior research shows that people who are high on CU traits associate with delinquent youth [32], and this was borne out in our zero-order correlations. Fite and Colder [31] found only reactive aggression to be related to peer delinquency in their longitudinal study (9–12 years of age), however. Discrepancies between our findings and Fite and Colder [31] could reflect our use of person-centred techniques, which create a combined reactive/proactive group or the age-cohort differences across the two studies. Nevertheless, in the present study, the zero-order correlations showed positive associations between both proactive and reactive aggression and peer delinquency.

Surprisingly, the groups were not differentiated on resistance to peer influence. Although resistance to peer influence was negatively correlated with reactive and proactive aggression, it did not differentiate the groups from each other, possibly indicating that it was less important than the individual-level and other peer measures in the model. As prior research has found [67], resistance to peer influence, in the present study, was associated with increases in chronological age. A further significant correlation was found between CU traits and resistance to peer influence, which suggested that greater levels of CU traits were related to less resistance to peer influence. Research has been equivocal in showing the influence of peers in relation to CU traits [68,69]. However, it could be that girls with CU traits experience pressure to conform to peers due to their heightened emotional reactivity [5].

There are several limitations that must be considered when interpreting our findings. Although some of our measures included investigator calculations, like gender composition of the peer group, an aim hidden from participants, all measures included self-report questionnaires. This may inflate relationships among measures due to shared method variance. Furthermore, it may be that self-reports of peer delinquency reflect a bias toward beliefs that others in our peer network are similar to us. Indeed, these two measures were highly correlated. Yet, research using self-reported peer delinquency and peer-reported delinquency both show similar relations with CU traits [32,56]. Further, the present study was a cross-sectional survey in a community sample, and so, we were not able to determine causal directions among our measures. We have attempted to interpret our findings with regard to multiple directions of effects, using prior research to guide our rationale. However, longitudinal research is needed to determine how these processes unfold over time. Thus, we would urge further research on social contextual measures over time.

The present study benefitted from a number of strengths that make our results applicable to the growing body of research on subtypes of aggression. We surveyed a large sample of girls with a varied age range from a community sample in Cyprus. We included multiple measures of behaviour, as well as social context using well-validated measures from both the developmental literature and the literature on abnormal behaviour. Many of these measures have been previously validated within Cypriot cohorts of boys and girls [52], and we showed expected relations among the main study measures.

Our findings have clinical implications for aggressive behaviour in girls. In line with research by Marsee and colleagues [2], our findings suggest that relational aggression may be important to assess in girls. That is, girls who use retaliatory and proactive functions of aggression may be particularly at risk for CU traits, which in prior research have been related to greater delinquency, aggression and risky behaviour [70]. Therefore, the presence of reactive/proactive relational aggression in girls may be a signal to practitioners looking to provide interventions for aggression and antisocial behaviour. With regard to reactions to parenting, Tilton-Weaver and colleagues [36] showed that CU traits were related to greater perception of parental overcontrol. Girls with CU traits and reactive/proactive relational aggression, then, may perceive parents as thwarting their autonomy. The reactions to perceiving overcontrol will be important to examine, since Tilton-Weaver and colleagues [36] found youths “closed down” communications with their parents. Indeed, longitudinal studies with those with elevated levels of CU traits show that parents become stressed and back off controlling their children when they exhibit CU traits accompanied by antisocial behaviour [17,71], and they have less knowledge about what their children are doing over time [72]. Therefore, interventions that target the parent-child relationship may be fruitful for keeping lines of communication open.

In sum, the present study supports the distinctiveness of aggression subtypes for adolescent girls’ relational aggression. In particular, we found support for distinguishing between the reactive aggressive and combined reactive/proactive aggressive groups when including social contextual facets and moving beyond individual maladaptive functioning. We found further support for the combined type of aggression in girls (and for relational aggression) to be specifically distinguished from the other groups by CU traits.

## 9. Conclusions

Adolescent girls perpetrate aggression by ruining their relationships with peers; they may gossip, exclude others or get others to start hating someone. Prior research suggested that although both girls and boys perpetrate relational aggression, the sequela for girls may be poor adjustment problems such as delinquent activity and peer relationships. It appears, then, that relational aggression—particularly when used for retaliatory as well as predatory functions—may be an important cue to a need for intervention for girls. We found that girls who used high levels of reactive and proactive relational aggression showed low levels of caring and empathy toward others, characteristics associated with CU traits. Moreover, when considering aggression in females, we argue it is important to take into account peer dynamics and the parent-child relationship. Our findings suggest those girls who show relational aggression for the purpose of gaining status and revenge feel restrained by their parents and may gravitate toward female relationships that support their behaviour.
